# Longitudinal evaluation of the association between Insulin-like growth factor-1, Bone specific alkaline phosphatase and changes in mandibular length

**DOI:** 10.1038/s41598-019-48067-7

**Published:** 2019-08-09

**Authors:** Tulika Tripathi, Prateek Gupta, Priyank Rai, Jitender Sharma, Vinod Kumar Gupta, Navneet Singh, Mahesh Verma

**Affiliations:** 1 Department of Orthodontics and Dentofacial Orthopaedics, Maulana Azad Institute of Dental Sciences, Bahadur Shah Zafar Marg, New Delhi, 110002 India; 2 Department of Biochemistry, Govind Ballabh Pant Institute of Postgraduate Medical Education and Research, Jawaharlal Nehru Marg, New Delhi, 110002 India; 3 Maulana Azad Institute of Dental Sciences, Bahadur Shah Zafar Marg, New Delhi, 110002 India

**Keywords:** Hydrolases, Predictive markers, Prognostic markers

## Abstract

The aim of the current longitudinal study was to assess the levels of serum Bone-specific alkaline phosphatase (BALP) and serum Insulin like growth factor-1 (IGF-1) in different cervical vertebral maturation index (CVMI) stages and observe their association with the mandibular growth. Blood samples and lateral cephalograms of 63 subjects (age group of 11–17 years) were obtained at two time points, 12 months apart. On the basis of CVMI, all subjects were divided into six groups based on whether the subjects remained in same CVMI stage or transitioned to the next CVMI stage. Annual mandibular length was related with serum BALP and serum IGF-1 levels estimated using ELISA. Serum IGF-1 and BALP attained highest levels at CVMI stage 3 with peak BALP levels observed earlier than IGF-1. Although a positive correlation was determined between IGF-1 and BALP but BALP followed skeletal growth pattern more precisely. Overall IGF-1 and BALP were negatively correlated with mandibular length with notable growth in CVMI groups 3–3 (P < 0.01), 3-4 (P < 0.01), 4-4 (P < 0.001) and 5-5 (P < 0.001). In conclusion, BALP is a potential biomarker for skeletal growth assessment. However, the mandibular growth pattern was independent of changes in IGF-1 and BALP.

## Introduction

Identification of skeletal pattern and residual growth potential entails precise association of the growth and development with the maturational stage of each individual in order to decide a proper treatment plan^1^. Mandibular growth is subjected to substantial contemplation as this bone enlarges the most during adolescence owing to presence of cartilaginous portion of the mandibular condyle, which grows maximally in the craniofacial complex^2–4^. Thus, determination of peak mandibular growth velocity is fundamental in effectuating greatest impact of functional/orthopedic appliances with concomitant reduction in the demands made on changes in tooth position and to predict post treatment occlusal stability^5,6^.

**Figure 1:**
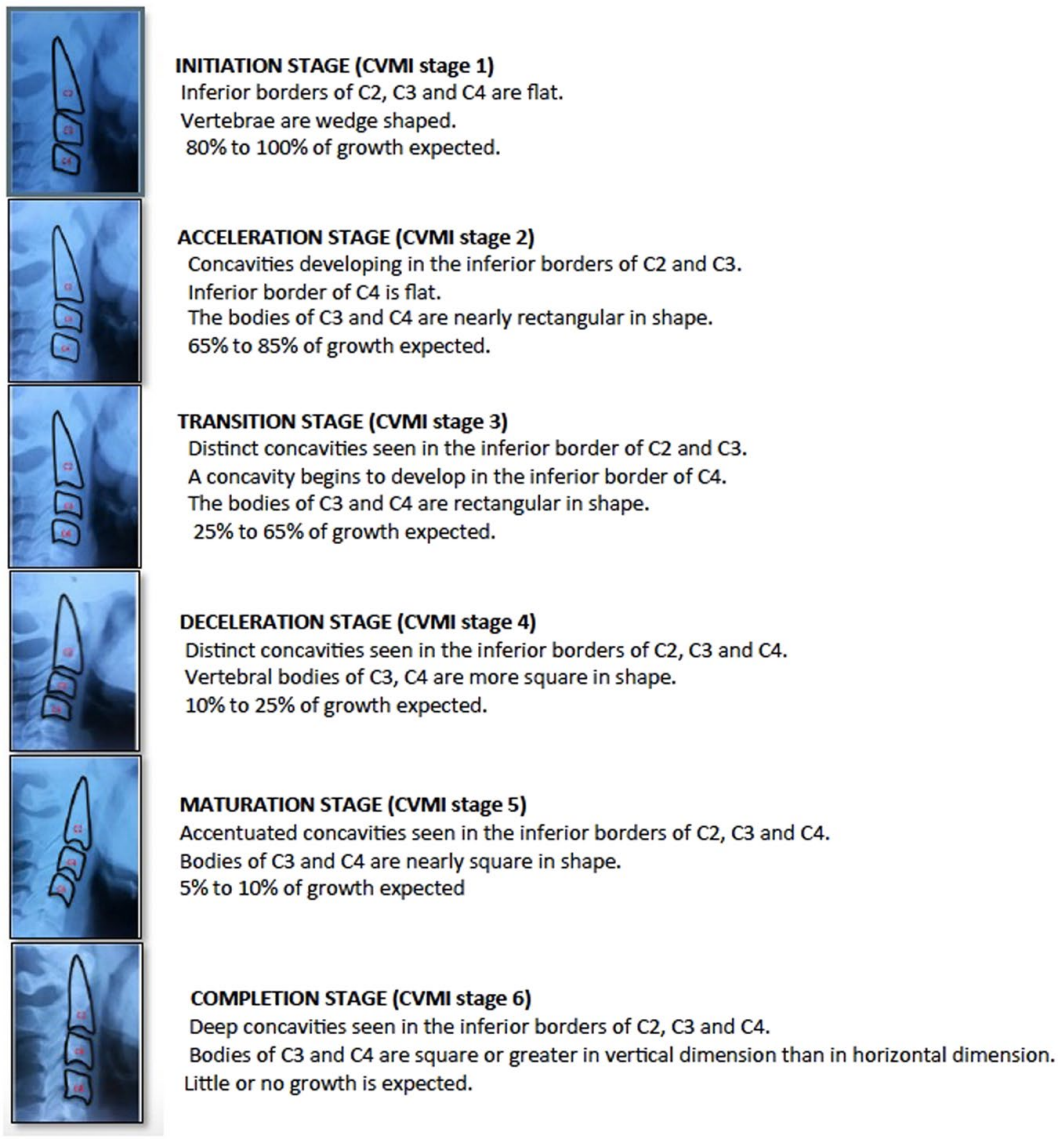
Hassel and Farman’s Cervical vertebral maturation index (CVMI) stages.

Cervical vertebral maturation index (CVMI) is the contemporary method which allows for appraisal of skeletal maturation on the basis of the morphology of second to fourth cervical vertebrae visualized on lateral cephalogram. There are six stages (Fig. 1), which correspond to the amount of expected growth in an individual with peak growth in stages 3 and 4. However, radiographic exposure, variability in subjective assessment of the radiographs and unsuitability in predicting intensity of mandibular growth spurt are some of the limitations, which affect the reliability of CVMI^7–9^. Moreover, cervical vertebrae morphological assessment is difficult due to variability in cervical area of vertebral column in different skeletal relationships of the jaws, body posture and shapes of facial components. Further, second and third cervical vertebrae were found to be fused in 14.3% of healthy subjects^10–14^.

Assessment of biochemical markers, signified as molecular representatives of the process of skeletal growth, possibly predict bone growth more accurately^15^. Insulin like growth factor-1 (IGF-1) is present in bone matrix, which even localizes to mandibular condylar cartilage and expedites growth process under growth hormone and androgens^16,17^. Bone-specific alkaline phosphatase (BALP) is the foremost component in the course of calcification process of the matrix^18,19^. and exhibits increase in levels during adolescence^20^. Since promising results of BALP and IGF-1 in depicting skeletal growth has been observed in cross sectional studies in regard to concordance with CVMI^21–27^, we ventured into longitudinal assessment of the changes in serum IGF-1 and BALP during active growth period and further study the relationship between these biomarkers and mandibular length.

## Results

### Baseline data

This longitudinal study comprised of sixty-three subjects which were segregated into three CVMI stages at the time of induction of the subjects in the study (T1); CVMI stage 3 (n = 19), CVMI stage 4 (n = 23) and CVMI stage 5 (n = 21). After 1 year follow up (T2), these subjects were categorized again according to their CVMI status; CVMI stage 3 (n = 9), CVMI stage 4 (n = 30), CVMI stage 5 (n = 20) and CVMI stage 6 (n = 4). The CVMI groups were formulated by further subdivision depending on whether the subjects remained in same CVMI stage or transitioned to the next CVMI stage viz. 3-3(n = 9), 3-4(n = 10), 4-4(n = 20), 4-5(n = 3), 5-5(n = 17) and 5-6(n = 4).

### Interexaminer and intraexaminer agreement

Interexaminer and intraexaminer kappa statistics for both weeks showed good agreement for T1 (0.88 and 0.95 respectively) and T2 (0.88 and 0.92 respectively). Mandibular length intraclass correlation coefficient was 0.998.

### Comparison of IGF-1 and BALP at T1 and T2

Tables 1 and 2 shows that mean IGF-1 and BALP levels at T1 were statistically significant in stage 4 (P < 0.05) and stage 3 (P < 0.01) respectively as compared to other stages. At T2, mean IGF-1 and BALP levels were highest in stage 3. BALP levels were significantly lower at stage 5 as compared to stage 3 (P < 0.05) and 4 (P < 0.01).

**Table 1:** Descriptive statistics for serum IGF-1, serum BALP and mandibular length (mm) in each CVMI stage at T1 and T2.

			IGF-1 (ng/ml)				BALP (ng/ml)				Mandibular length (mm)			
CVMI	N	Age	Range		Mean ± SD	Median	Range		Mean ± SD	Median	Range		Mean ± SD	Median
**At T1**														
3	19	13.85 ± 1.56	230.00	600.00	458.78 ± 136.59	500.00	17.00	236.00	143.94 ± 56.37	150.00	93.24	114.84	103.96 ± 5.77	103.70
4	23	15.07 ± 1.63	157.50	600.00	532.71 ± 130.11	600.00	38.00	199.00	95.52 ± 47.45	74.00	93.01	114.50	104.56 ± 5.73	103.10
5	21	16.01 ± 1.99	250.00	600.00	380.52 ± 97.35	365.00	17.00	221.00	72.33 ± 44.02	53.00	92.60	120.97	105.29 ± 6.76	104.46
**At T2**														
3	9	14.97 ± 1.81	225.00	600.00	516.94 ± 132.62	600.00	28.50	160.50	96.88 ± 50.87	88.00	95.33	115.41	104.76 ± 6.98	104.79
4	30	15.64 ± 1.56	130.00	600.00	414.31 ± 171.45	494.50	17.50	165.50	79.71 ± 41.80	70.50	95.35	115.33	106.67 ± 4.96	106.43
5	20	17.15 ± 2.15	157.50	600.00	394.77 ± 160.53	362.50	12.00	144.00	49.62 ± 31.44	36.50	92.69	122.04	105.55 ± 7.14	104.75
6	4	17.49 ± 0.75	125.00	245.00	204.87 ± 55.09	224.75	18.50	126.50	76.87 ± 47.65	81.25	103.80	113.55	108.63 ± 5.11	108.59

**Table 2:** Comparison among CVMI stages for serum IGF-1, serum BALP and mandibular length at T1 and T2 using Mann-Whitney U test.

		IGF-1		BALP		Mandibular length	
**CVMI stage**							
a	b	a-b	P value	a-b	P value	a-b	P value
**At T1**							
3	4	−100.00	0.024*	76.00	0.006*	0.60	0.752
	5	135.00	0.075	97.00	0.001*	−0.76	0.507
4	5	235.00	0.001*	21.00	0.062	−1.36	0.664
**At T2**							
3	4	105.50	0.076	17.50	0.405	−1.64	0.424
	5	237.50	0.073	51.50	0.042*	0.04	0.850
	6	375.25	0.011*	6.75	0.355	3.80	0.355
4	5	132.00	0.912	34.00	0.003*	1.68	0.452
	6	269.75	0.024*	−10.75	1.000	−2.16	0.392
5	6	137.75	0.016*	−44.75	0.313	−3.84	0.278

IGF-1: Insulin like growth factor-1; BALP: Bone-specific alkaline phosphatase; CVMI: cervical.

vertebral maturation index. *P < 0.05.

### Intra and intergroup comparison of IGF-1

**Figure 2:**
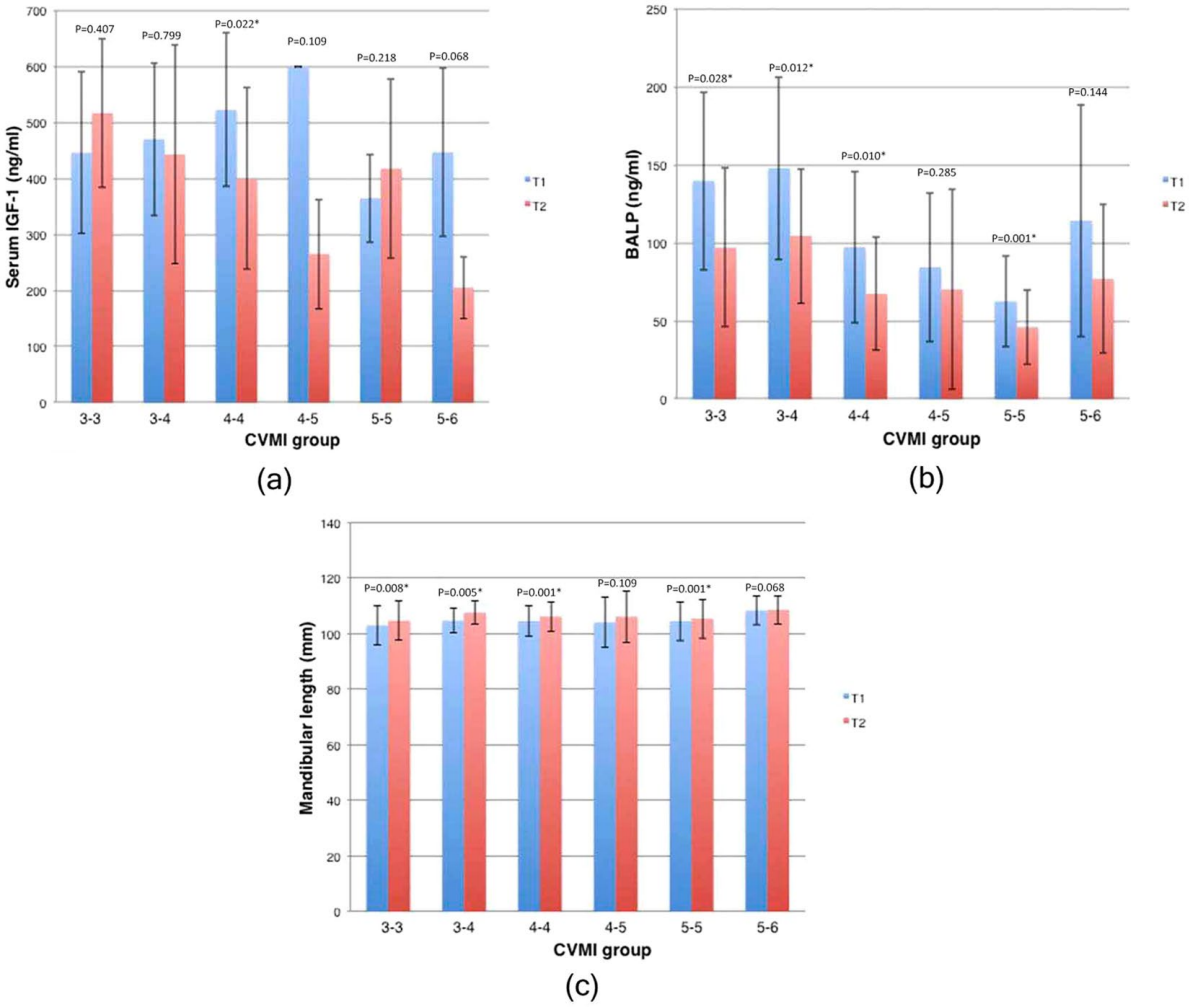
Bar graph comparing mean serum IGF-1 levels (**a**), BALP levels (**b**) and mandibular lengths (**c**) at T1 and T2 in different CVMI groups. (**a**) IGF-1 level is highest in CVMI group 3-3 at T2 and statistically significant between T1 and T2 (P < 0.05) at group 4-4. (**b**) BALP level is highest in CVMI group 3-3 at T1 and statistically significant between T1 and T2 at CVMI groups 3-3 (P < 0.05), 3-4 (P < 0.05), 4-4 (P < 0.01) and 5-5 (P < 0.001). (**c**) Mandibular growth is highest in CVMI group 3-4 and statistically significant at CVMI groups 3-3 (P < 0.05), 3-4 (P < 0.05), 4-4 (P < 0.001) and 5-5 (P < 0.001) between T1 and T2.

Table 3 shows IGF-1 values at T1 and T2 with Wilcoxon signed rank test for comparison at two time points in 6 groups. IGF-1 values exhibits increase in groups 3-3 and 5-5 and decrease in groups 3-4, 4-5 and 5-6 which were not found to be significant. IGF-1 values showed significant annual decrease in group 4-4 (P < 0.05). This has been graphically represented in Fig. 2(a). Our results (Table 4) also exhibit that increase in IGF-1 levels were not significantly different between groups 3-3 and 5-5.

**Table 3:** Descriptive statistics of serum IGF-1, BALP and mandibular length and Wilcoxon signed rank test for comparing their mean ranks between T1 and T2 at each CVMI group.

			T1				T2				P value (T2-T1)
Group	N	Age	Range		Mean ± SD	Median	Range		Mean ± SD	Median	
**IGF-1**											
3-3	9	14.97 ± 1.81	250.00	600.00	446.05 ± 144.07	482.00	225.00	600.00	516.94 ± 132.62	600.00	0.407
3-4	10	15.14 ± 1.37	230.00	600.00	470.25 ± 136.21	525.00	147.50	600.00	443.00 ± 195.11	575.00	0.799
4-4	20	15.23 ± 1.49	157.50	600.00	522.62 ± 137.04	600.00	130.00	600.00	399.97 ± 161.81	454.50	0.022*
4-5	3	15.94 ± 2.89	600.00	600.00	600.00 ± 0	600.00	172.50	368.00	265.16 ± 98.14	255.00	0.109
5-5	17	17.37 ± 2.03	250.00	528.00	364.91 ± 78.81	350.00	157.50	600.00	417.64 ± 160.29	365.00	0.218
5-6	4	17.49 ± 0.75	257.50	600.00	446.87 ± 151.01	465.00	125.00	245.00	204.87 ± 55.09	224.75	0.068
**BALP**											
3-3	9	14.97 ± 1.81	52.00	202.00	139.66 ± 57.08	164.00	28.50	160.50	96.88 ± 50.87	88.00	0.028*
3-4	10	15.14 ± 1.37	17.00	236.00	147.80 ± 58.52	148.00	17.50	160.00	104.45 ± 43.18	105.00	0.012*
4-4	20	15.23 ± 1.49	38.00	199.00	97.20 ± 48.41	75.00	20.50	165.50	67.35 ± 36.07	56.50	0.010*
4-5	3	15.94 ± 2.89	50.00	139.00	84.33 ± 47.85	64.00	30.50	144.00	70.16 ± 64.00	36.00	0.285
5-5	17	17.37 ± 2.03	17.00	112.00	62.47 ± 28.99	52.00	12.00	100.00	46.00 ± 23.85	37.00	0.001*
5-6	4	17.49 ± 0.75	49.00	221.00	114.25 ± 74.44	93.50	18.50	126.50	76.87 ± 47.65	81.25	0.144
**Mandibular length**											
3-3	9	14.97 ± 1.81	93.24	114.84	103.08 ± 7.10	100.57	95.33	115.41	104.76 ± 6.98	104.79	0.008*
3-4	10	15.14 ± 1.37	98.82	113.54	104.76 ± 4.51	104.30	101.00	115.33	107.61 ± 4.25	107.41	0.005*
4-4	20	15.23 ± 1.49	93.01	114.50	104.63 ± 5.44	103.26	95.35	115.20	106.20 ± 5.32	104.73	0.001*
4-5	3	15.94 ± 2.89	96.69	114.06	104.10 ± 8.96	101.54	98.08	116.27	106.15 ± 9.26	104.10	0.109
5-5	17	17.37 ± 2.03	92.60	120.97	104.57 ± 7.02	104.46	92.69	122.04	105.44 ± 7.05	104.97	0.001*
5-6	4	17.49 ± 0.75	103.72	113.52	108.36 ± 5.10	108.09	103.80	113.55	108.63 ± 5.11	108.59	0.068

IGF-1: Insulin like growth factor-1; BALP: Bone-specific alkaline phosphatase; CVMI: cervical vertebral maturation index. *P < 0.05.

**Table 4:** Mann-Whitney U test comparing each group for mean ranks of annual change in serum IGF-1, serum BALP and mandibular length.

		Group a versus b		
		IGF-1	BALP	Mandibular length
Group a	Group b	P value	P value	P value
3-3	3-4	0.462	0.513	0.165
	4-4	0.045*	0.346	0.164
	4-5	0.013*	0.405	0.229
	5-5	0.936	0.186	0.978
	5-6	0.021*	0.877	0.279
3-4	4-4	0.391	0.194	0.271
	4-5	0.128	0.091	0.866
	5-5	0.563	0.008*	0.029*
	5-6	0.257	0.479	0.024*
4-4	4-5	0.091	0.437	0.315
	5-5	0.010*	0.437	0.012*
	5-6	0.163	0.587	0.005*
4-5	5-5	0.007*	0.958	0.039*
	5-6	0.289	0.480	0.034*
5-5	5-6	0.005*	0.369	0.106

IGF-1: Insulin like growth factor-1; BALP: Bone-specific alkaline phosphatase. *P < 0.05.

### Intra and intergroup comparison of BALP

Comparison of BALP values between T1 and T2 using Wilcoxon signed rank test in 6 groups are presented in Table 3 and graphically depicted in Fig. 2(b). Decrease in BALP values was observed in all groups with significant difference in groups 3-3 (P < 0.05), 3-4 (P < 0.05), 4-4 (P < 0.01) and 5-5 (P < 0.001) (Table 4).

### Intragroup and intergroup comparison of mandibular length

Significant increase in mandibular lengths in groups 3-3 (P < 0.01), 3-4 (P < 0.01), 4-4 (P < 0.001) and 5-5 (P < 0.001) is depicted in Table 3 and Fig. 2(c). However, increase in mandibular length in group 5-5 differed significantly from other stages (P < 0.05) except groups 3-3 and 5-6 as shown in Table 4.

### Correlation among annual percentage changes in serum IGF-1, BALP and mandibular length

Spearman correlation coefficient depicted an overall negative correlation for annual percentage changes in serum IGF-1 and BALP with mandibular length. However, computation of correlation in separate groups revealed positively correlated IGF-1 and mandibular length in groups 4-4 and 5-5 while BALP and mandibular length in group 5-6. Annual percentage changes in IGF-1 and BALP exhibited a positive correlation across all groups except group 5-5 where a negative correlation was observed (Table 5).

**Table 5:** Spearman correlation among annual percentage change in values of serum IGF-1, serum BALP and mandibular length.

		IGF-1 vs mandibular length		BALP vs mandibular length		IGF-1 vs BALP	
Group	N	Spearman correlation	P value	Spearman correlation	P value	Spearman correlation	P value
3-3	9	−0.550	0.125	−0.167	0.668	0.183	0.637
3-4	10	−0.596	0.069	−0.200	0.580	0.261	0.466
4-4	20	0.153	0.520	−0.063	0.791	0.340	0.142
4-5	3	−0.500	0.667	−0.500	0.667	1.000	0.001*
5-5	17	0.280	0.276	−0.085	0.746	−0.086	0.742
5-6	4	−0.200	0.800	0.200	0.800	0.600	0.400
Overall	63	−0.129	0.314	−0.037	0.771	0.159	0.213

IGF-1: Insulin like growth factor-1; BALP: Bone-specific alkaline phosphatase. *P < 0.05.

## Discussion

The present longitudinal study was carried out in subjects having CVMI stages 3, 4, and 5 as these stages signify pubertal and post pubertal period of growth which is highly relevant for treatment planning in orthodontics. Based on the observations of Hassel and Farman^28^, 65% of adolescent growth is expected in these stages and positive results of growth modification therapy have been observed. Moreover, majority of the patients who reported for orthodontic treatment belonged to these cervical stages.

Various biomarkers have been explored in earlier studies for their association with skeletal growth^29^. In our earlier cross-sectional studies, we assessed the relationship of different biomarkers namely IGF-1, BALP and osteocalcin with skeletal growth estimated using CVMI. However, correlation was found to be significant for IGF-1 and BALP with skeletal maturity across CVMI stages^26,27^. Hence, we conducted a longitudinal study employing IGF-1 and BALP to determine their relationship with mandibular growth through different CVMI stages.

In the present study, CVMI groups were formulated as some subjects remained in the same CVMI stage after one year follow up while others progressed to next CVMI stage. With a change from CVMI stages 3 to 6, the growth potential decreases and ceases at stage 6.

In the present investigation, peak IGF-1 levels were seen in stage 4 CVMI at T1 and in stage 3 CVMI at T2 which was similar to previous studies^22,23,25–27,30,31^ whereas Masoud *et al*.^21^ reported peak IGF-1 levels at stage 5 CVMI.

After one year follow up, IGF-1 levels were raised in groups 3-3 and 5-5 while reduced in 3-4, 4-4, 4-5 and 5-6 groups. This indicated that peak IGF-1 levels occur in CVMI stage 3 and decline thereafter but rises again at 5-5. This may be attributed to late occurrence of peak in muscle growth as compared to bone growth and both are under the regulation of IGF-1^32^.

BALP followed a similar pattern at both T1 and T2 with peak levels in stage 3 which corresponded to the outcome of study by Tripathi *et al*.^27^. The results of our study also strengthened the results of previous studies where peak alkaline phosphatase levels were observed in pubertal phase of skeletal maturation in comparison to post pubertal phases^33–35^. Prospective analysis precisely localized the peak BALP levels at an earlier point in CVMI stage 3 which descend during the advanced stage 3. Thus, the results divulged that BALP levels peak and fall earlier than IGF-1, although CVMI stage remained same. Further, the peak levels of IGF-1 at CVMI stage 3 corresponded with highest facial growth as observed by Fishman^36^ and Hassel and Farman^28^. Moreover, Masoud *et al*.^21^ reported that 25% to 65% of growth during adolescence also correlated with stage 3 CVMI signifying greater role of biomarkers in revealing skeletal maturation status. BALP levels slumped significantly in transition groups 3-3, 3-4, 4-4 and 5-5. Moreover, rise in IGF-1 levels in group 5-5 endorse an assertive association of BALP with skeletal maturation as compared to IGF-1.

Mandibular length increased significantly during CVMI groups 3-3, 3-4, 4-4 and 5-5 with highest levels (2.84 mm) observed in 3-4 similar to findings by Franchi *et al*.^37^. This might be due to greatest bone apposition at condylion and maximum mandibular growth at cervical stage 3-4 as observed by Gu *et al*.^38^. BALP and IGF-1 levels declined during the same groups except during group 5-5 when IGF-1 levels increased. This depicted a growth of mandible even during the declining levels of these biomarkers. It does not annul the importance of IGF-1 and BALP in mandibular growth rather signifying that these are not the major factors regulating mandibular growth. The least mandibular growth increment at group 5-6 in our study also corroborated to the Gu *et al*. findings of least bone apposition at condylion. The significant increase in mandibular length across different groups in our study can be attributed to the relative constancy in the deposition at posterior condylion throughout cervical stages as also suggested by Gu *et al*.^38^.

Mandibular growth observed during the transition group 4-5 was close to peak growth (2.05 mm) which might be due to pronounced deposition at superior condylion during this stage^38^. However, it was not found to be statistically significant which might be due to low sample size (n = 3) in this group. Thus, mandible continued to show significant growth during the CVMI stages 3 to 5. Annual mandibular growth rate was statistically insignificant between different groups which corresponded to the observations of Gomes and Lima^1^ except group 5-5 in our study.

Mandible is connected to the masticatory muscles, the nasomaxillary complex, the basicranium, salivary glands and all components of the servosystem^39,40^. Expansive changes and functional actions of these contiguous tissues actuate osteogenic membranes and cartilages throughout mandible to cause modifications and remodeling in all aspects to adapt to the changes due to growth and the altered functions of the soft tissues^39^. Further, maximum bite force progressively rises between 7 to 17 years of age^41^. Miyazaki *et al*.^42^ and Enomoto *et al*.^43^ observed that optimal force of the masticatory muscles is required for normal growth of the mandible in rats. Thus the increase in muscle mass and bite force might be a possible reason for continuous growth of mandible throughout stages 3-5 in the present study.

Annual percentage change in the values of IGF-1 and BALP was positively correlated with each other in different groups except group 5-5 when IGF-1 levels increase and BALP levels fall. Thus, the BALP and IGF-1 can be widely acknowledged as prospective biomarkers for growth assessment.

Annual change in IGF-1 and BALP exhibited an overall negative correlation with annual mandibular growth increment denoting that IGF-1 and BALP levels did not bear substantial association with increase in mandibular length after CVMI stage 3 while simultaneously not negating the importance of rise in IGF-1 and BALP levels in mandibular growth. This corresponds to the observations of Masoud *et al*. where significant negative correlation of IGF-1 and mandibular length was reported during descending patterns in females^44^. Mitani and Sato^8^ proposed that pubertal growth factors did not influence each part of the body simultaneously. They observed that body height, cervical vertebra and hand bone showed significant correlation in total growth whereas mandibular size has an independent regulation of these parameters, hence, corroborating with the present study in which the two biomarkers did not exhibit a positive correlation with mandibular growth.

Such antithetical observations after CVMI stage 3 can also be attributed to mandibular spatial position and histological composition. Mandibular condyle is exposed to various forces during movements of the jaw^45^. Histological framework of the condylar cartilage and its response to various forces and humoral factors is different from other cartilaginous tissues^46^. Condylar cartilage is the secondary cartilage that forms after the fetal period as compared to primary cartilage. The primary cartilage growth is influenced by growth hormones (general factor) and mechanical devices are capable of altering only the direction not the amount of growth. However, the condylar cartilage growth is determined not only by growth hormones but also local factors, being regulated by mechanical devices both in amount and direction of growth^47^. Lewis *et al*.^48^ demonstrated individual variability in the growth patterns and response of various bones brought about by alteration in the levels of circulating hormones.

Mandible derived mesenchymal stem cells show higher osteogenic potential to other skeletal bones which is attributed to greater amount of collagen and lower amount of mature crosslinks giving it the properties of immature bone with low degree of mineralization as compared to other long bones^49,50^. These alongwith the observation that trabecular bone formation in subcondylar region is unaffected with age, mandibular condylar cartilage bears prolonged growth potential^51^. Thus, IGF-1 and BALP markers may not be solely responsible for the continuous growth of the mandible through the stages 3 to 5.

BALP, a bone specific marker has been longitudinally investigated and compared with radiographic growth assessment indicator for the first time. Further, it is the foremost correlation of BALP with mandibular length and another growth marker, IGF-1 in a prospective study. Moreover, gender difference in BALP might be investigated to reveal its specific role during growth process. Since a definitive correlation has been found between CVMI and these biochemical markers, a longer follow up with a larger sample size can be done to derive reference charts. These charts will facilitate precise estimation of growth in an individual requiring growth modification therapy especially in cases where CVMI fails to clearly delineate the exact growth status. The precise evaluation of growth status forms the basis of growth modification therapy in routine orthodontic practice and is essential as it reduces the burden for more complex treatment involving extractions and orthognathic surgeries. This will facilitate simplification of therapy and conservation of resources.

In conclusion, we observed that serum BALP followed the skeletal growth pattern with peak level at an earlier point in CVMI stage 3 and after progression of one year, it showed descent in advanced stage 3. In group 3-3, serum IGF-1 attained peak levels which descended with change in CVMI stage but ascended again in group 5-5. Overall a positive correlation was found between IGF-1 and BALP across CVMI stages except in group 5-5. IGF-1 and BALP negatively correlated with mandibular length across CVMI stages 3 to 5 except in group 5-5 between IGF-1 and mandibular length. Our study has highlighted that BALP is superior to IGF-1 in corresponding to bone growth pattern as determined by CVMI and might be an adjunct to contemporary skeletal growth indicators to reveal the precise growth status. Mandibular growth has not been observed to be solely governed by biomarkers. There are other intrinsic (genetic) and extrinsic (environmental) factors viz. respiration, swallowing pattern and posture, which influence mandibular growth and need investigation to better elucidate the pattern of mandibular growth.

## Methods

### Sample

This present study was longitudinal in which 78 North Indian subjects in the age group of 11–17 years were recruited. The subjects were selected from the patients who visited the Department of Orthodontics and Dentofacial Orthopaedics for treatment. Ethical committee of Maulana Azad Institute of Dental Sciences approved the study (reference no. MAIDS/2014). Study was conducted and all methods were performed following the relevant guidelines and regulations. Bilingual patient information sheet was used to explain the research procedure to the subjects and their parents. Further, informed consent was signed by the parent (for his/her child participation in the study) for collection of blood samples twice at 12-month interval (T1 and T2 time points). Subjects having CVMI stage 3, 4 or 5 with no growth disturbance, systemic disease, bleeding anomaly, history of long term medication, trauma or surgical intervention in the cervical vertebral region were included in the study. In order to exclude the effects of ethnicity, socioeconomic level and family demographics on growth pattern, subjects which corresponded to height and weight standards according to age by the growth charts of the Indian Academy of Pediatrics were included in the study. Out of the seventy eight subjects registered for the study, 10 did not report for follow up and five patients refused to undergo subsequent blood sampling. Hence, this study reports data of 63 individuals with complete records at both T1 and T2. These subjects were further subdivided into 6 groups at T2 time point to better elucidate the relationship between CVMI status and biomarker levels. Lateral cephalograms of all the patients were taken in natural head position using Planmeca proline XC cephalostat (Finland) with the teeth in centric occlusion and were evaluated according to Hassel and Farman^28^ method (Fig. 1) at both T1 and T2. Each radiograph was labeled with the patient code to anonymise, following which two examiners (TT and PG) independently assessed all the radiographs for inter-examiner reliability. Both the examiners also examined the radiographs after a week for intra-examiner reliability.

The following procedure was performed at T1 and T2 for assessment of biomarker levels and mandibular length.

### Blood sample collection

Peripheral venous blood sample (five mililitres) was collected for each subject. In order to avoid diurnal variations, blood sample was collected between 0900 and 1000 hours and the lateral cephalograms were taken at the same time. Calcium, phosphorous and alkaline phosphatase were also estimated from the collected blood to exclude subjects with bone metabolism disorder. Each serum sample obtained by centrifugation was pipetted equally in two different plastic Eppendorf tubes (Eppendorf, Hamburg, Germany) for estimation of IGF-1 and BALP respectively and kept at −80 °C preceding assay.

### Laboratory assays

All the samples and reagents were brought to room temperature at the time of performing assay. Serum IGF-1 and BALP were measured using sandwich ELISA with serum IGF-1 ELISA (DRG International, USA) and Microvue BALP ELISA (Quidel Corporation, CA, USA) kits respectively. Serum concentrations of IGF-1 and BALP were determined by using mean absorbance values on the plotted standard calibration curves.

### Mandibular length assessment

**Figure 3:**
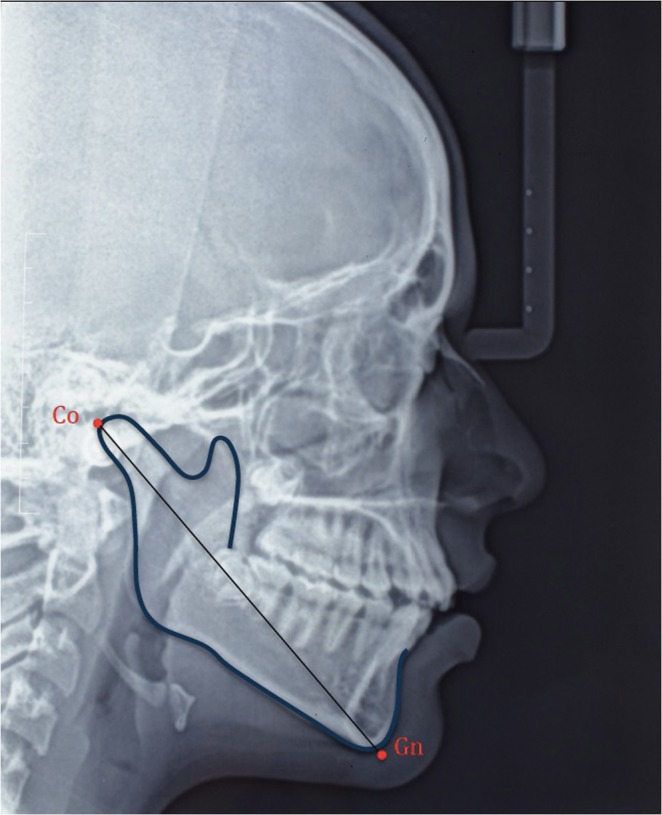
Mandibular length from Condylion (Co) to Gnathion (Gn).

The mandibular length was measured on tracings of the lateral cephalograms from Condylion (Co; most superior posterior point on the condyle) to Gnathion (Gn; most anterior inferior point on the hard tissue chin) (Fig. 3). The same author measured the mandibular length on an another tracing of the same radiograph after a week for intraexaminer reliability.

### Statistical analysis

SPSS software for Windows (version 23.0; SPSS, Chicago, Ill) was used to perform statistical analysis. A P value < 0.05 was considered statistically significant for all tests. Intra and inter-examiner reliabilities for CVMI staging at both T1 and T2 were measured using the weighted kappa statistic. Intraclass correlation coefficient was calculated for mandibular length to determine intraexaminer reliability. Skewness, kurtosis and Shapiro-Wilks W tests found that the data was not normally distributed. Comparison of mean ranks of serum IGF-1, serum BALP and mandibular length at one-year interval in different groups was performed using Wilcoxon signed rank test. The Mann–Whitney U test was employed to compare among different stages and also among different groups. Spearman correlation coefficients were calculated among serum IGF-1, serum BALP and mandibular length in each group.

## Data Availability

The data generated and analysed during the current study is available from the corresponding author on request.
